# Correction: Engineering of a chitin deacetylase to generate tailor-made chitosan polymers

**DOI:** 10.1371/journal.pbio.3003286

**Published:** 2025-07-15

**Authors:** Martin Bonin, Antonia L. Irion, Anika Jürß, Sergi Pascual, Stefan Cord-Landwehr, Antoni Planas, Bruno M. Moerschbacher

The Figs 2, 6 and 8 are in low resolution. Please view the Figs 2, 6 and 8 here.

**Fig 2:**
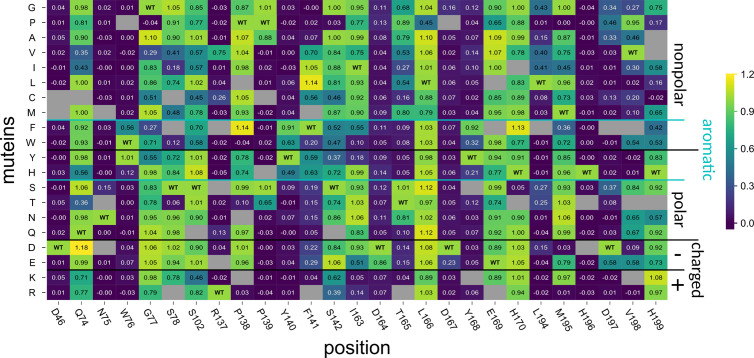
Activity of PesCDA muteins on A4 normalized to PesCDA^nm^ estimated by fluorescence-based quantification of free primary amines. Each column shows the activity of all available muteins at a given position, as indicated below the matrix. Missing muteins are grayed out. For orientation, PesCDAnm is included in each column at the corresponding position of the wild-type (WT) amino acid. The muteins are grouped by the properties—nonpolar, polar, charged (+/−), or aromatic—of the residue by which the WT amino acid was exchanged. Corresponding standard deviations can be found in a separate heatmap in S3 Fig (n = 3–4). All values can be found in S1 Data.

**Fig 6:**
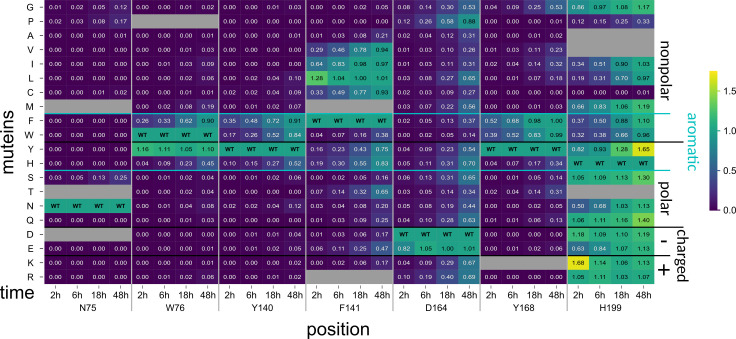
Subset of PesCDA muteins activity on A4 normalized to PesCDA^nm^ estimated by product detection via HPLC-MS. Each column shows the activity of all available muteins at one position for the indicated incubation time. Missing muteins are grayed out. For orientation, PesCDAnm is included in each column at the corresponding position of the wild-type (WT) amino acid. The muteins are grouped by their properties—nonpolar, polar, charged (+/−), and aromatic—of the residue by which the WT amino acid was exchanged. Corresponding standard deviations can be found in a separate heatmap in S4 Fig (n = 4). All values can be found in S3 Data.

**Fig 8:**
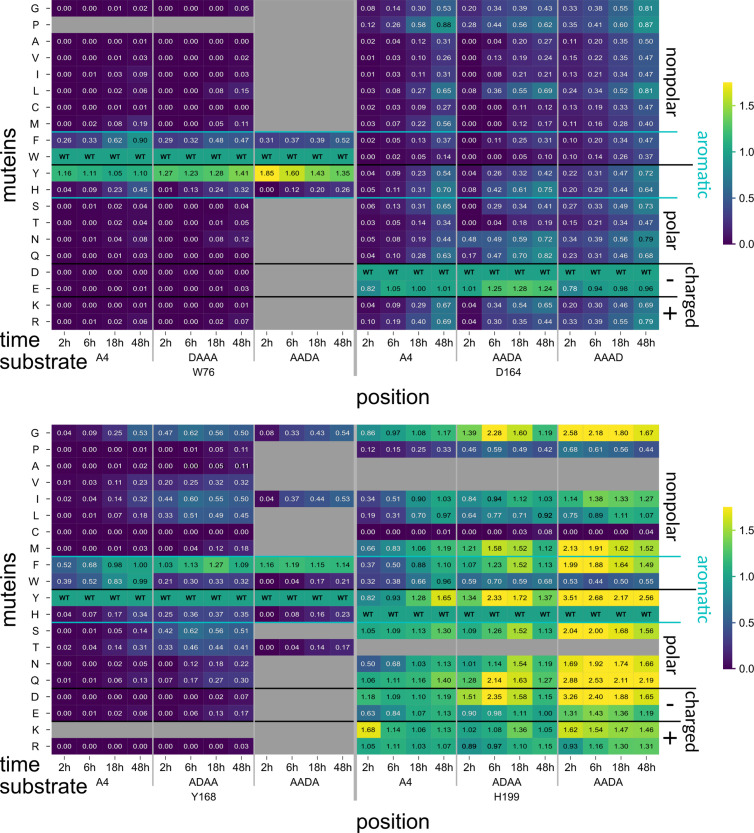
Subset of PesCDA muteins’ activity on A4 and different A3D1 substrates normalized to PesCDAnm estimated by product detection via HPLC-MS. Each column shows the activity of all tested muteins at one position for the indicated incubation time and substrate. Missing or not tested muteins are grayed out. For orientation, PesCDAnm is included in each column at the corresponding position of the wild-type (WT) amino acid. The muteins are grouped by their properties—nonpolar, polar, charged (+/−), and aromatic—of the residue by which the WT amino acid was exchanged. Corresponding standard deviations can be found in a separate heatmap in S5 Fig (n = 4). All values can be found in S3 Data.
