# Supplementary figures and images for: Engineering of a chitin deacetylase to generate tailor-made chitosan polymers
Source: PLoS Biol. 2024 Jan 18;22(1):e3002459. doi: 10.1371/journal.pbio.3002459 (PMC10796014; doi:10.1371/journal.pbio.3002459)

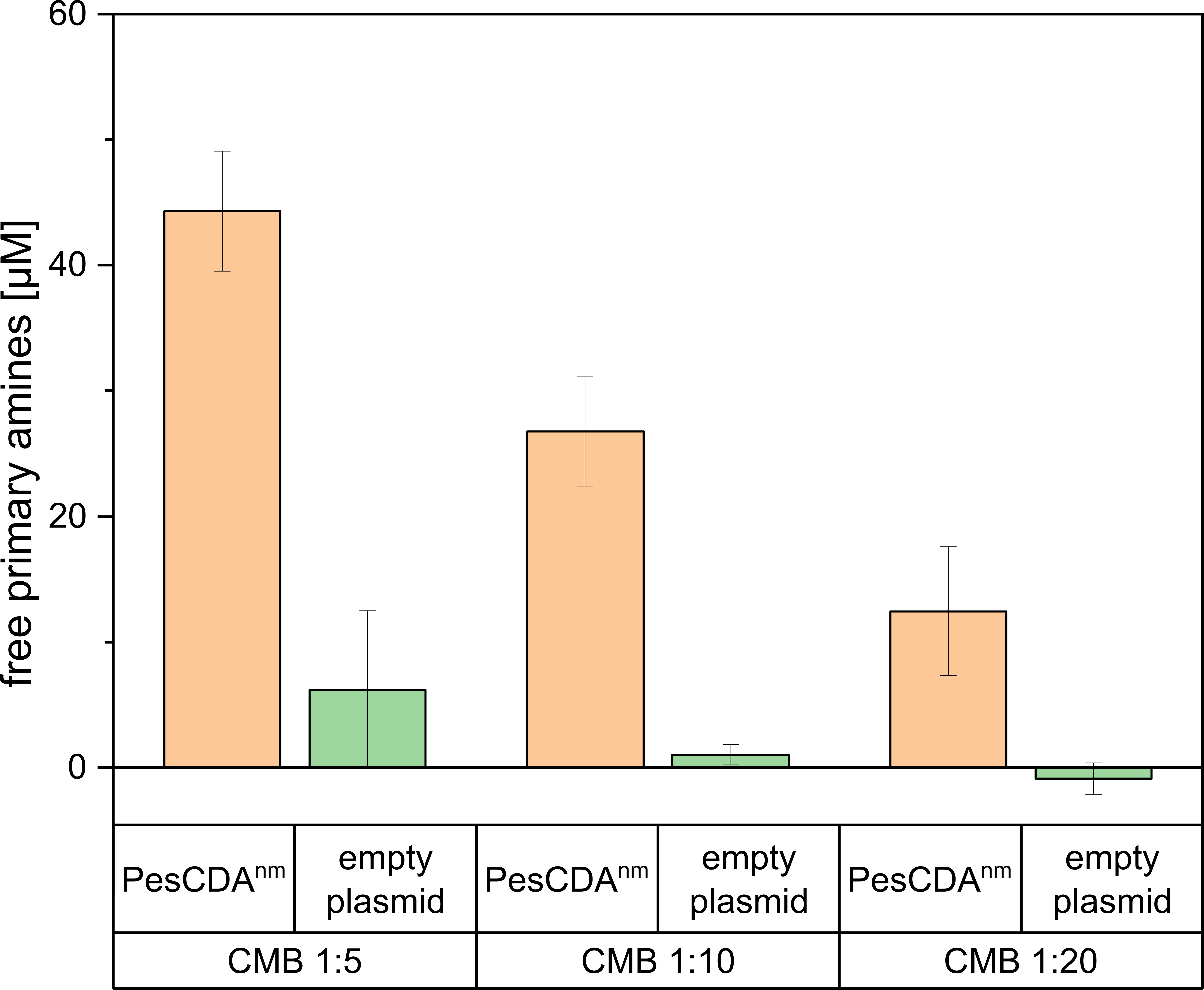

Supplement: S1 Fig — The PesCDAnm as well as an empty vector control were subjected to the fluorescamine-based screening. Using 3 glucosamine standards (15 μM, 50 μM, and 100 μM), the concentration of free primary amines were calculated. As expected, the empty plasmid control shows values close to 0, whereas PesCDAnm shows an increasing amount of free primary amines formed with a decreasing dilution of CMBs. With a strong activity increase still being visible comparing the 1:10 to the 1:5 dilution, we settled on the 1:10 dilution assuming that the CMBs are saturated with enzymes. The results further show that with all dilutions less then 10% of the 500 μM A4 substrates were deacetylated. All values can be found in S1 Data. (TIFF) [file pbio.3002459.s001.tiff]

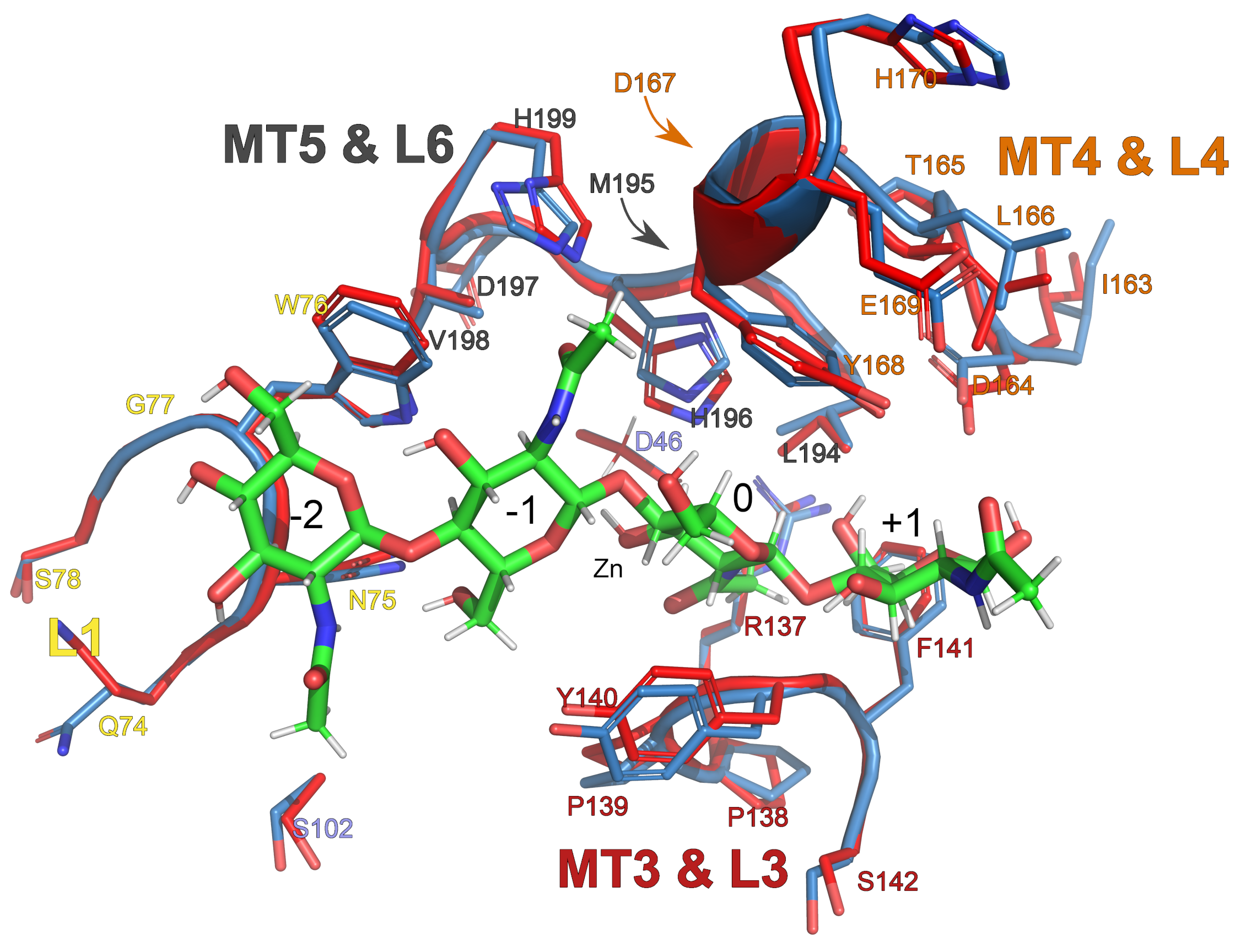

Supplement: S2 Fig — The AlphaFold2 model from the AlphaFold Protein Structure Database [35,36] was aligned to our SWISS-MODEL with an RMSD = 1.341 Å on all atoms and an RMSD = 0.782 Å on the 27 SSM library amino acids. Our SWISS-MODEL is shown with blue, and the AlphaFold2 model is shown with red carbon atoms, respectively. For an easier overall comparison, the other colors and labels are reused from Fig 1. (TIFF) [file pbio.3002459.s002.tiff]

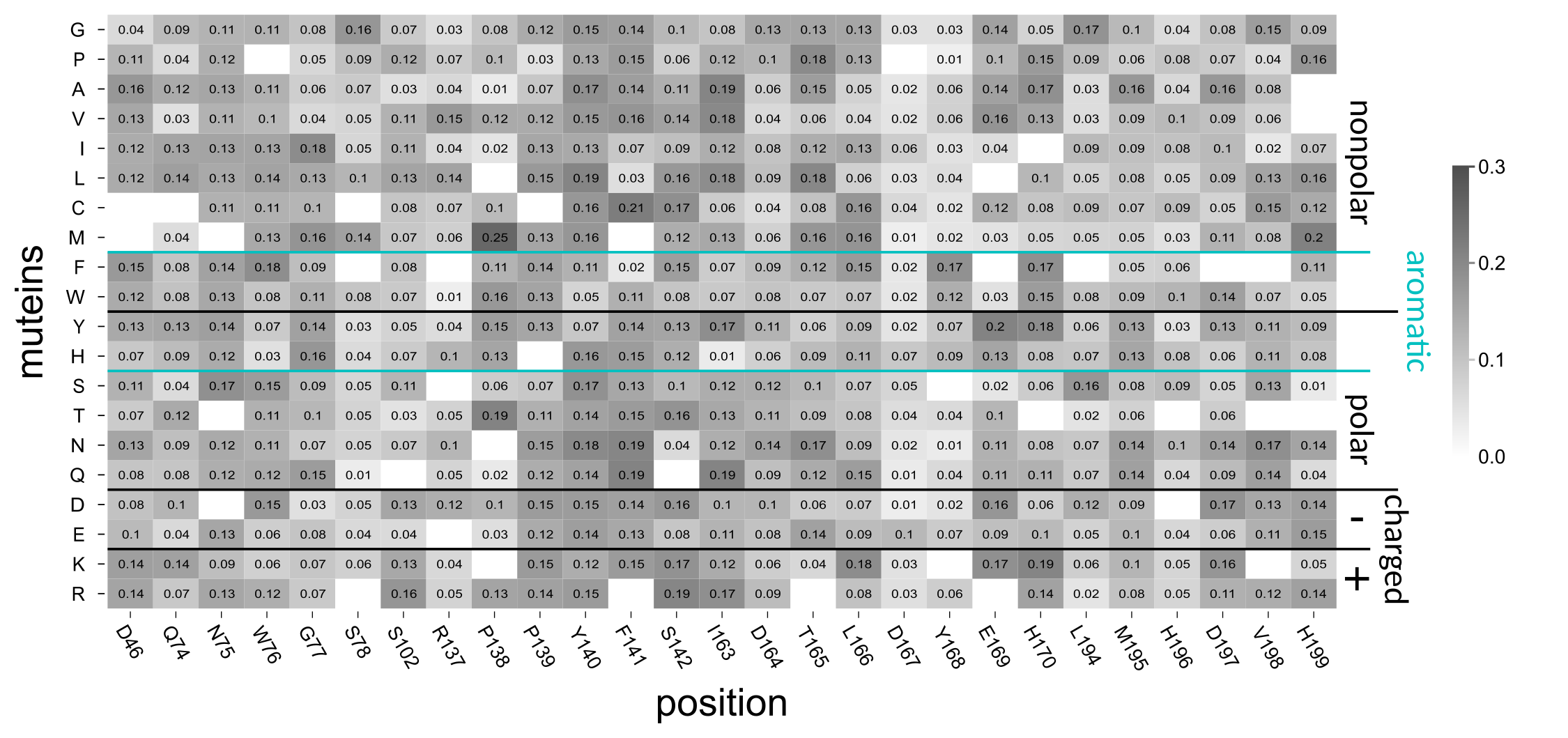

Supplement: S3 Fig — Each column shows the standard deviation of all available muteins at a given position, as indicated below the matrix. Empty fields indicate missing muteins. The muteins are grouped by the properties—nonpolar, polar, charged (+/−), or aromatic—of the residue by which the wild-type amino acid was exchanged (n = 3–4). All values can be found in S1 Data. (TIFF) [file pbio.3002459.s003.tiff]

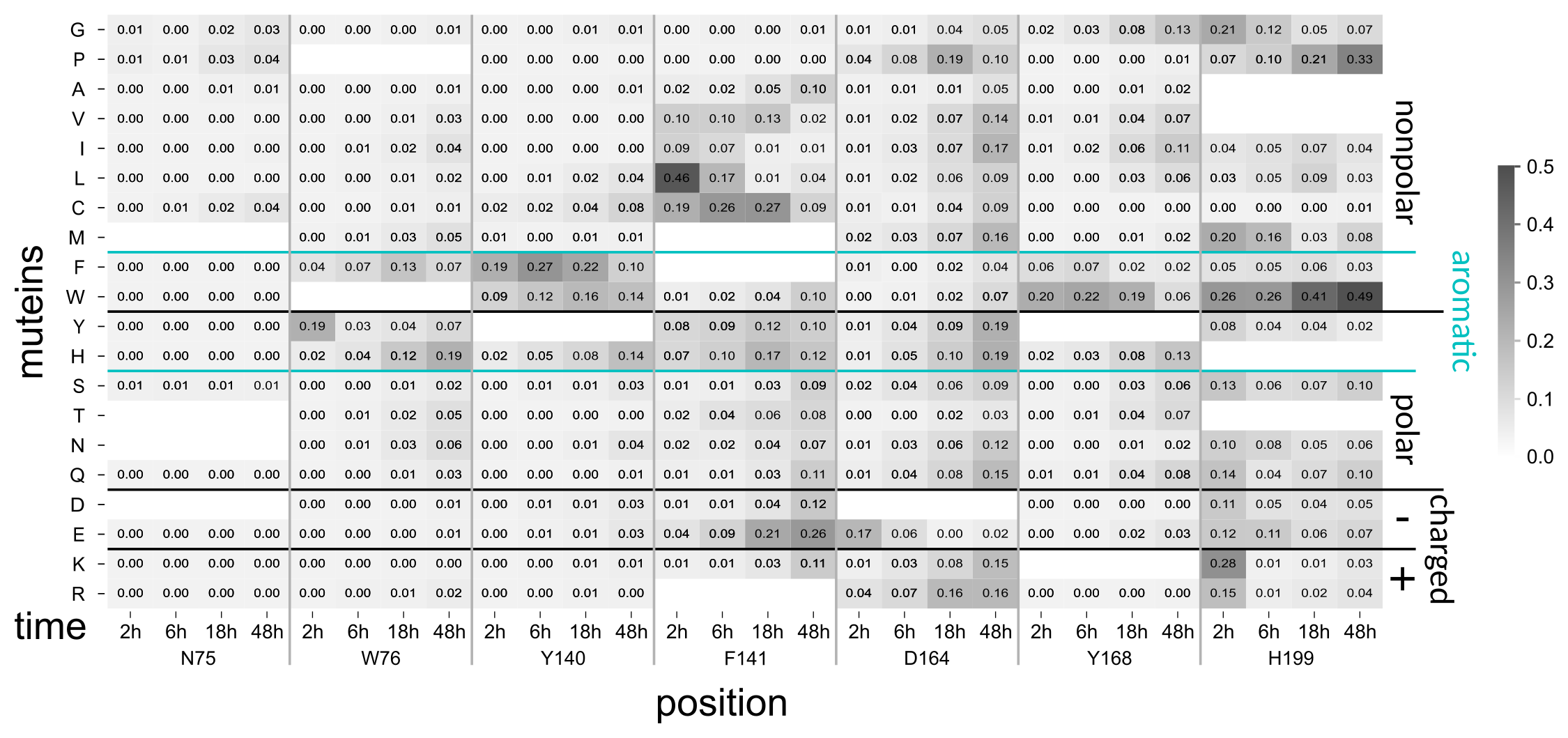

Supplement: S4 Fig — Each column shows the standard deviation of all available muteins at a given position, as indicated below the matrix. Empty fields indicate missing muteins. The muteins are grouped by the properties—nonpolar, polar, charged (+/−), or aromatic—of the residue by which the wild-type amino acid was exchanged (n = 4). All values can be found in S3 Data. (TIFF) [file pbio.3002459.s004.tiff]

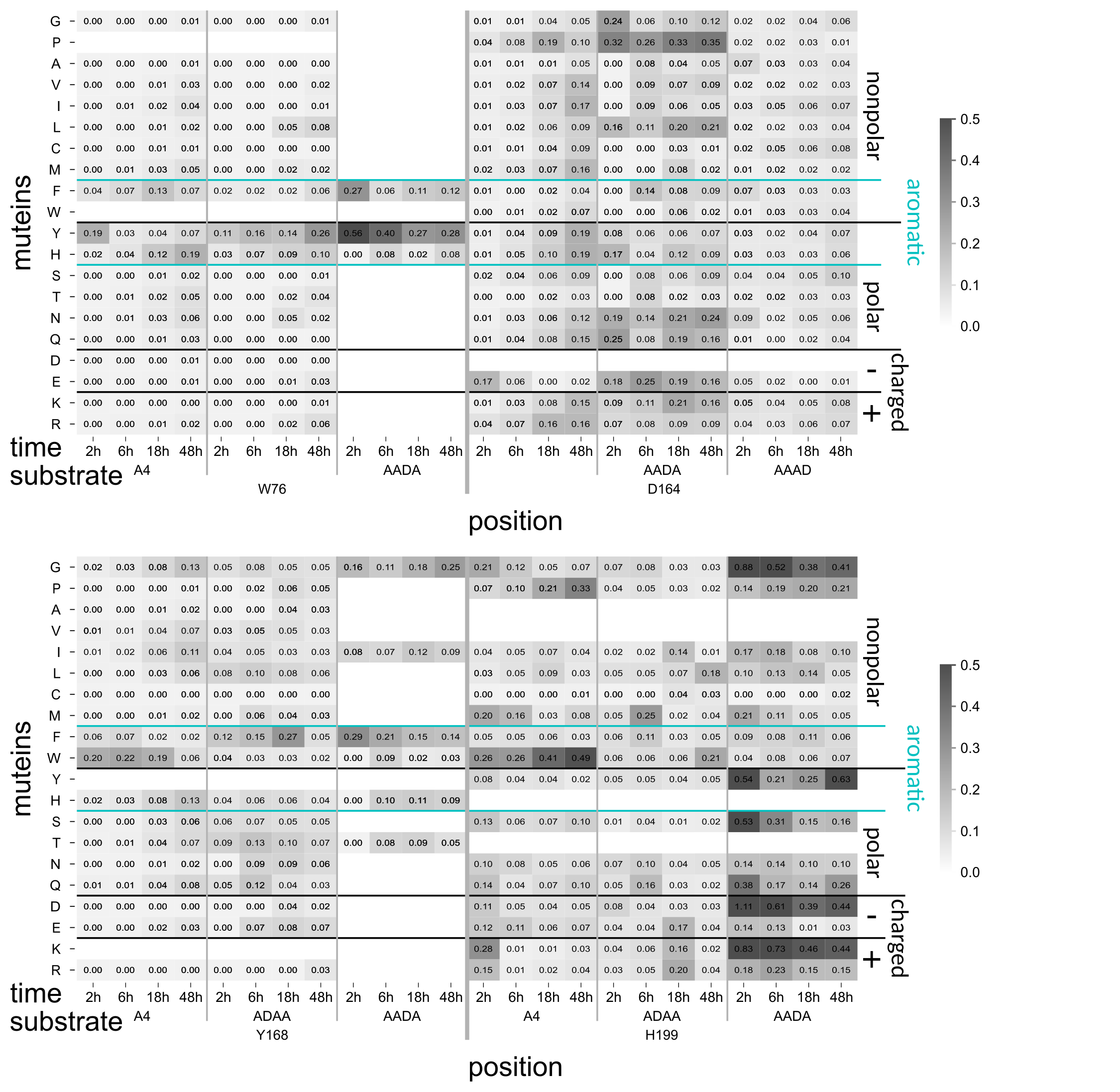

Supplement: S5 Fig — Each column shows the standard deviation of all available muteins at a given position, as indicated below the matrix. Empty fields indicate missing or not tested muteins. The muteins are grouped by the properties—nonpolar, polar, charged (+/−), or aromatic—of the residue by which the wild-type amino acid was exchanged (n = 4). All values can be found in S3 Data. (TIFF) [file pbio.3002459.s005.tiff]

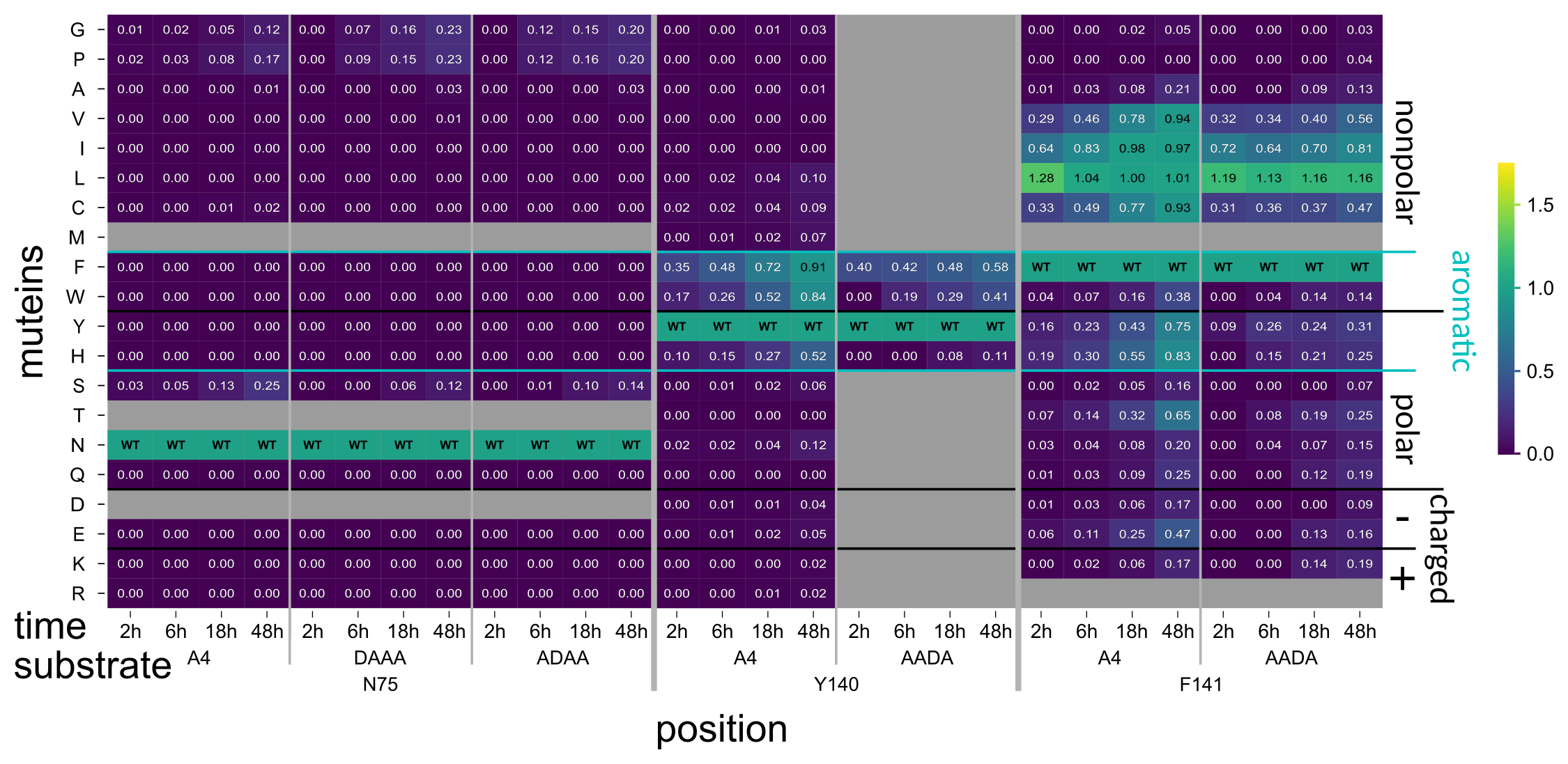

Supplement: S6 Fig — Each column shows the activity of all tested muteins at one position for the indicated incubation time and substrate. Missing or not tested muteins are grayed out. For orientation, the PesCDAnm is included in each column at the corresponding position of the wild-type (WT) amino acid. The muteins are grouped by their properties nonpolar, polar, charged (+/−), and aromatic. Corresponding standard deviations can be found in a separate heatmap in S7 Fig (n = 4). All values can be found in S3 Data. (TIFF) [file pbio.3002459.s006.tiff]

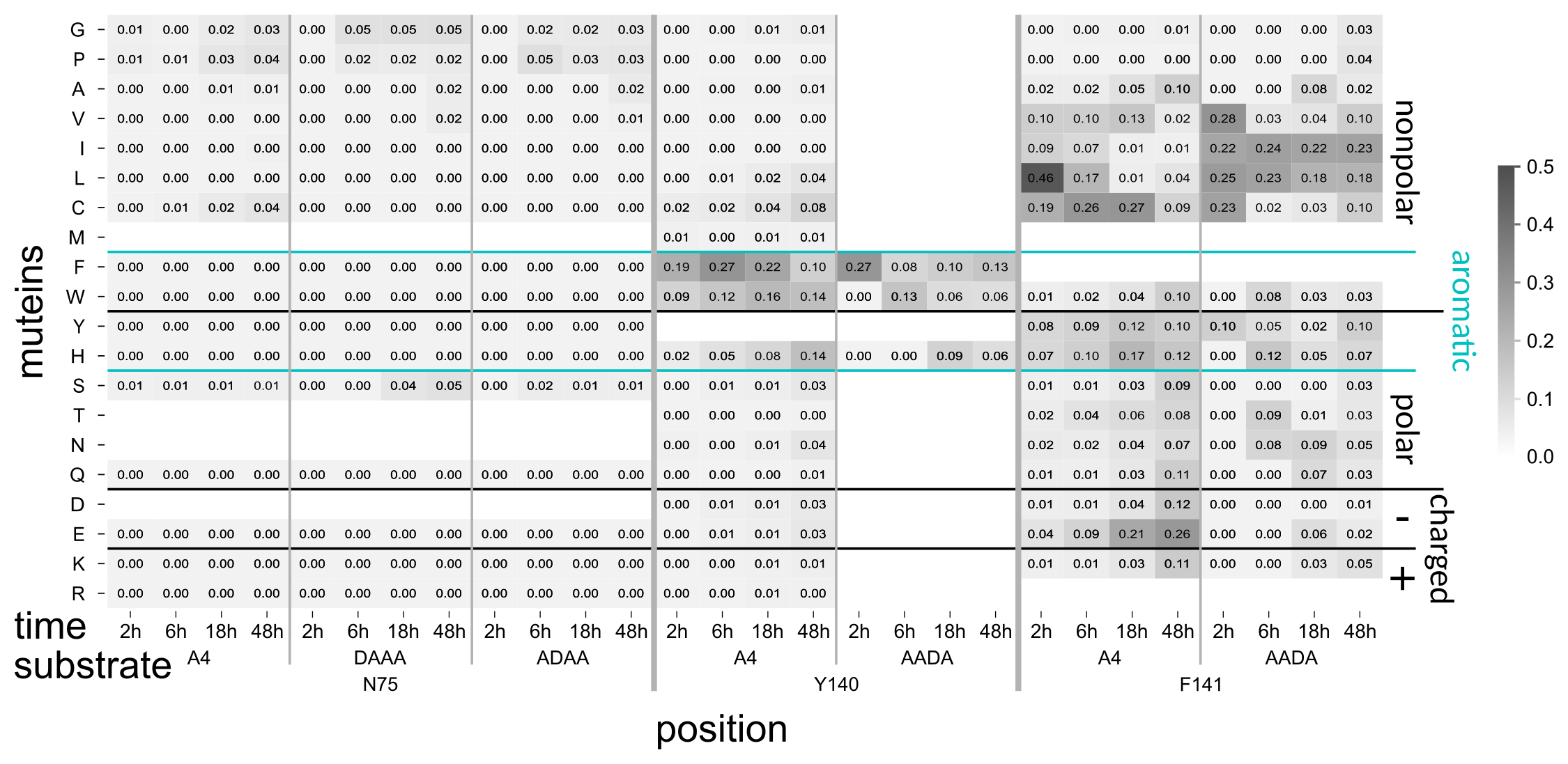

Supplement: S7 Fig — Each column shows the standard deviation of all available muteins at a given position, as indicated below the matrix. Empty fields indicate missing or not tested muteins. The muteins are grouped by the properties—nonpolar, polar, charged (+/−), or aromatic—of the residue by which the wild-type amino acid was exchanged (n = 4). All values can be found in S3 Data. (TIFF) [file pbio.3002459.s007.tiff]

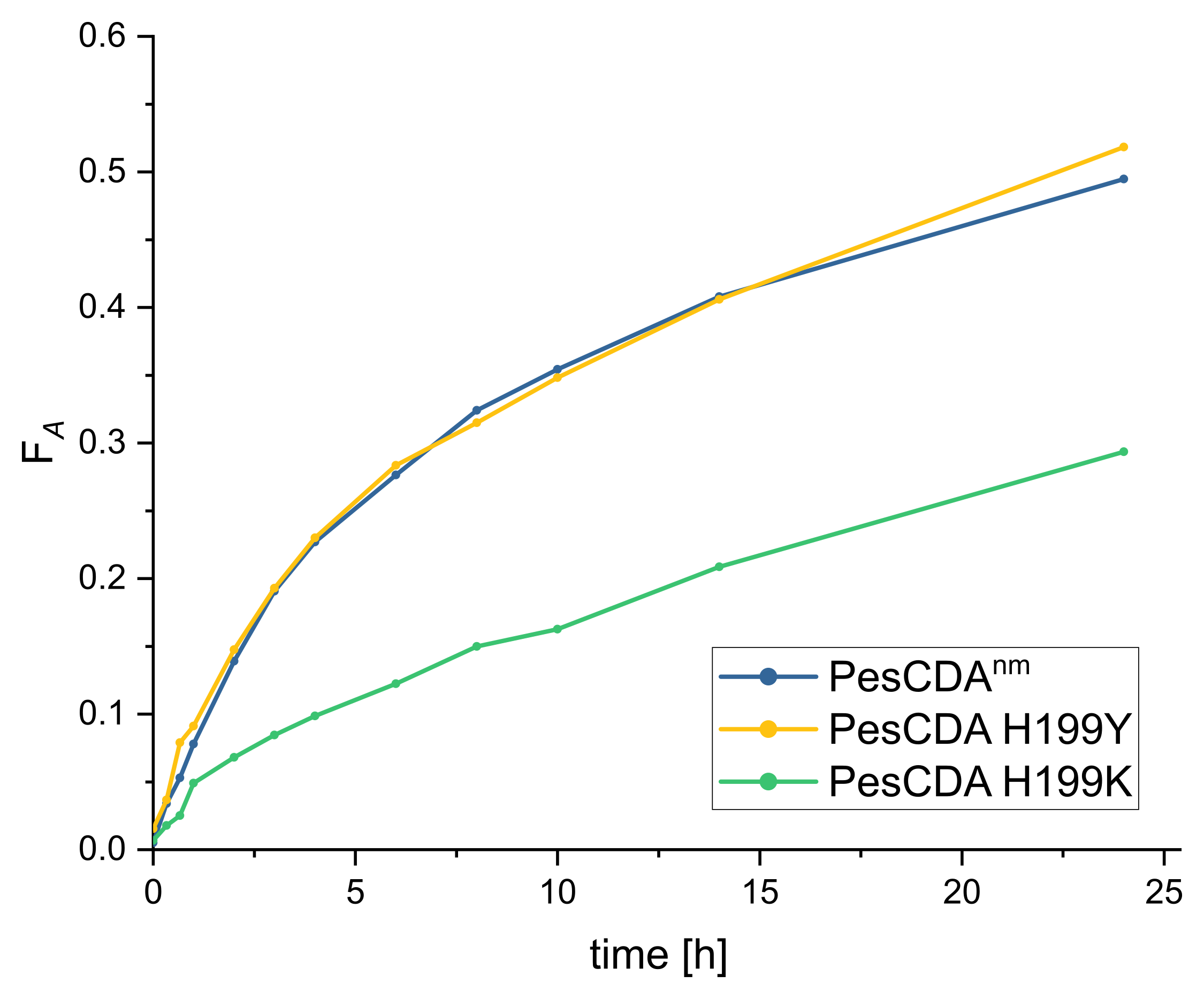

Supplement: S8 Fig — FA determined over time for N-acetylation of polyglucosamine using PesCDAnm, PesCDA H199Y, and PesCDA H199K. Samples analyzed here with FA’s close to 0.1, 0.2, 0.3, 0.4, and 0.5 were used for chitinosanase digestion to determine the PA of these chitosans (n = 1). All values can be found in S5 Data. (TIFF) [file pbio.3002459.s008.tiff]

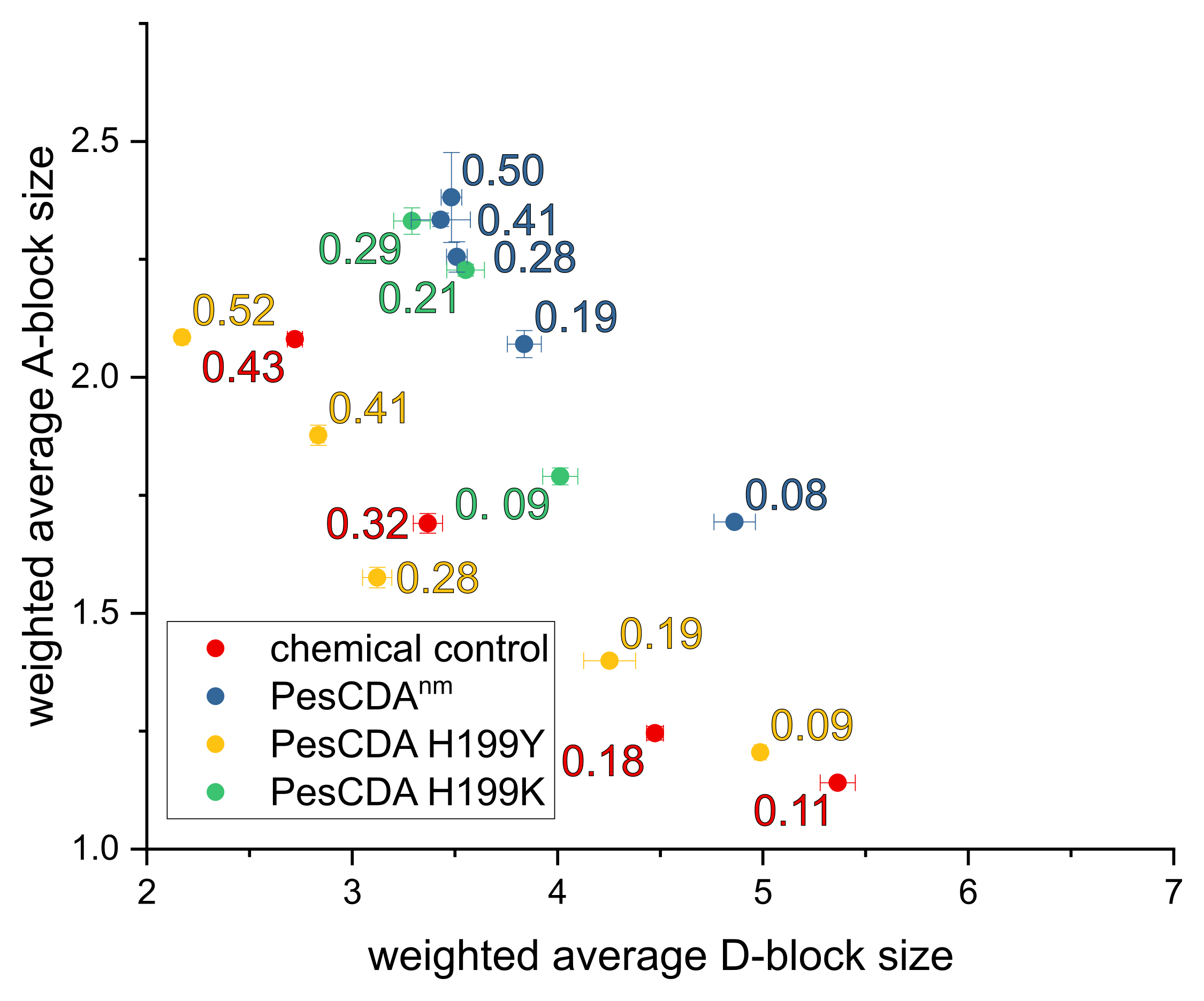

Supplement: S9 Fig — Obtained fragments after chitinosanase digestion were separated via hydrophilic liquid interaction chromatography (HILIC). For the corresponding figure measured via size exclusion chromatography, see Fig 9B (n = 3). All values can be found in S5 Data. (TIFF) [file pbio.3002459.s009.tiff]

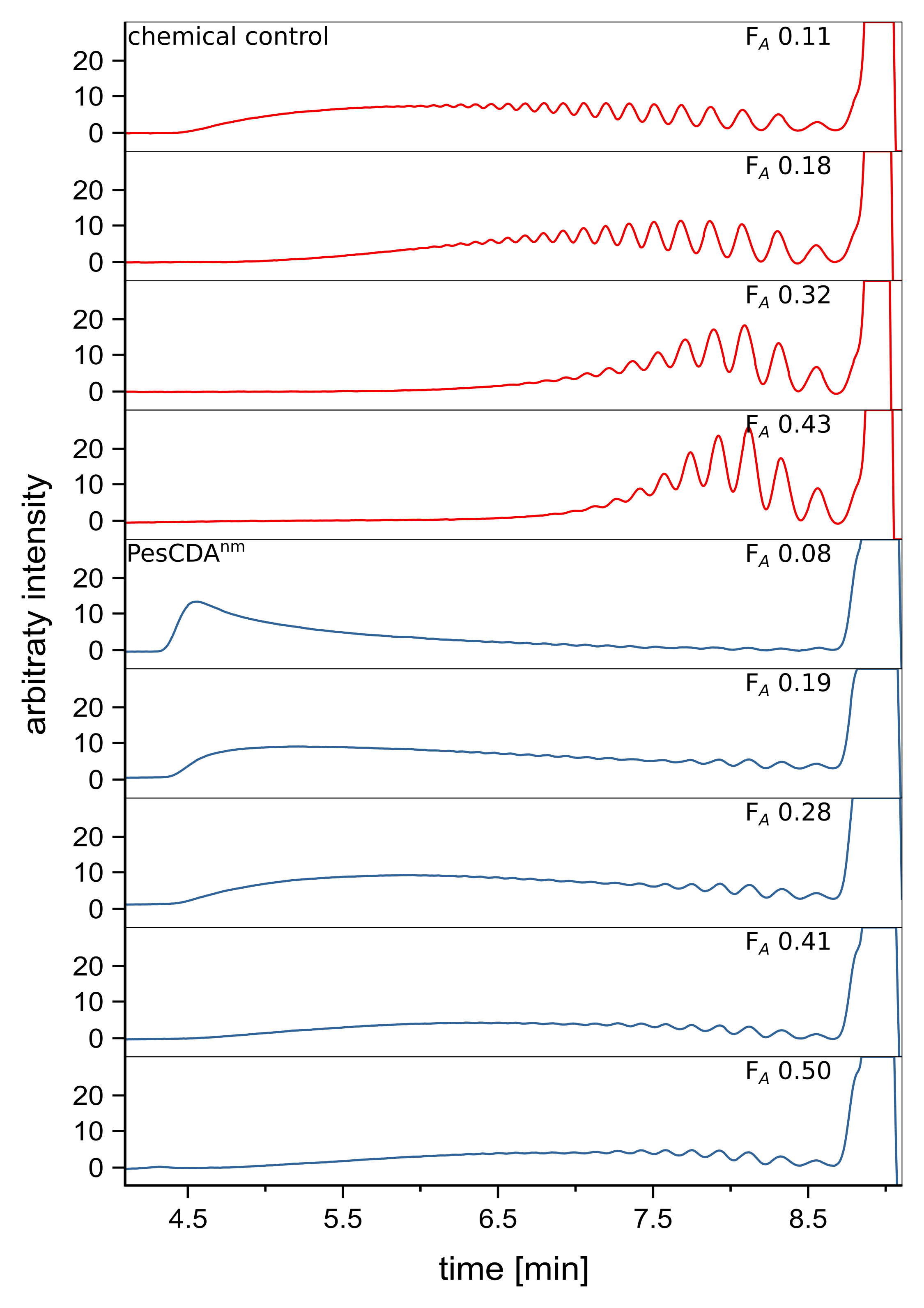

Supplement: S10 Fig — Samples were measured in triplicates. Here, an exemplary chromatogram is shown which best resembles all replicates. (TIFF) [file pbio.3002459.s010.tiff]

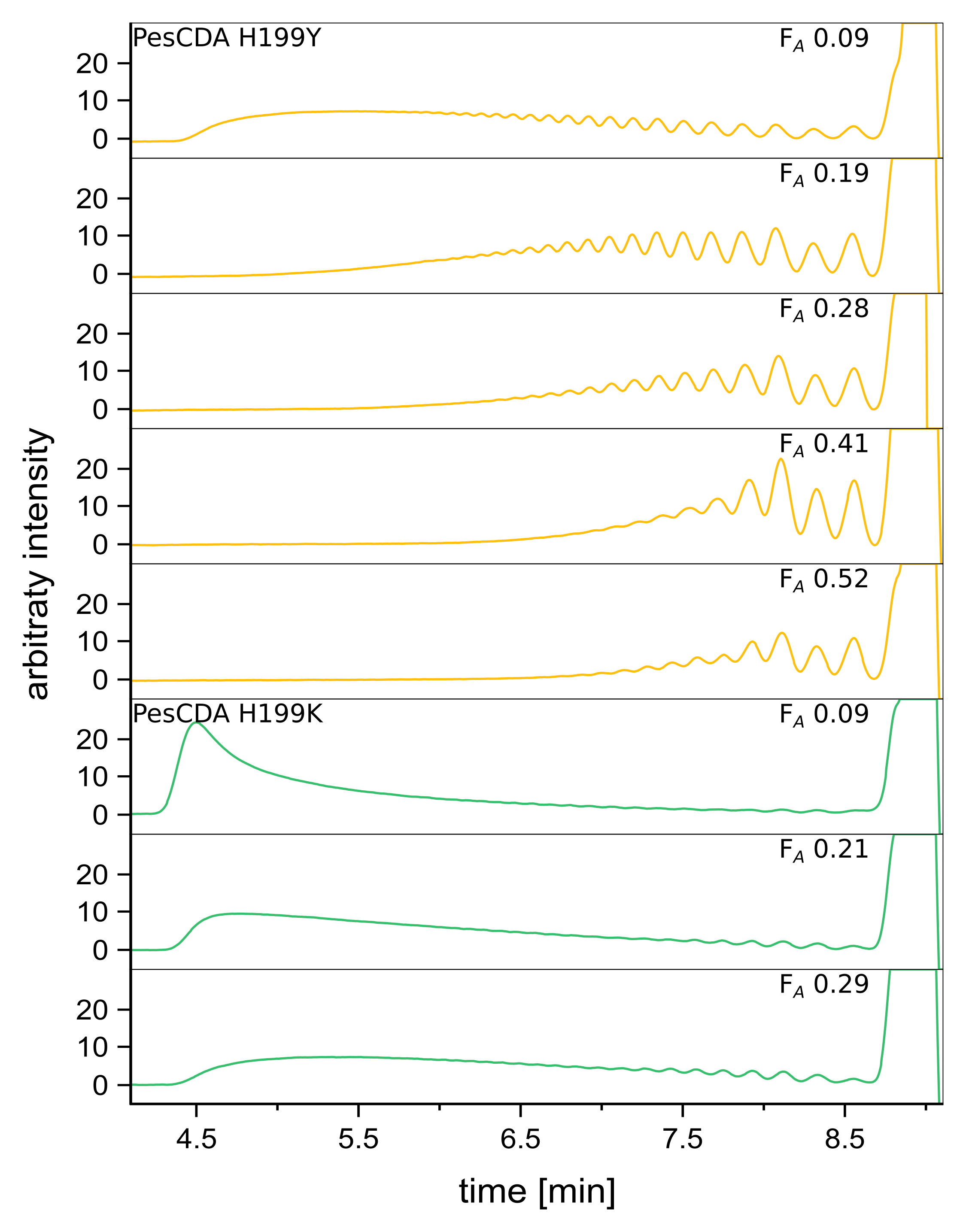

Supplement: S11 Fig — Samples were measured in triplicates. Here, an exemplary chromatogram is shown which best resembles all replicates. (TIFF) [file pbio.3002459.s011.tiff]

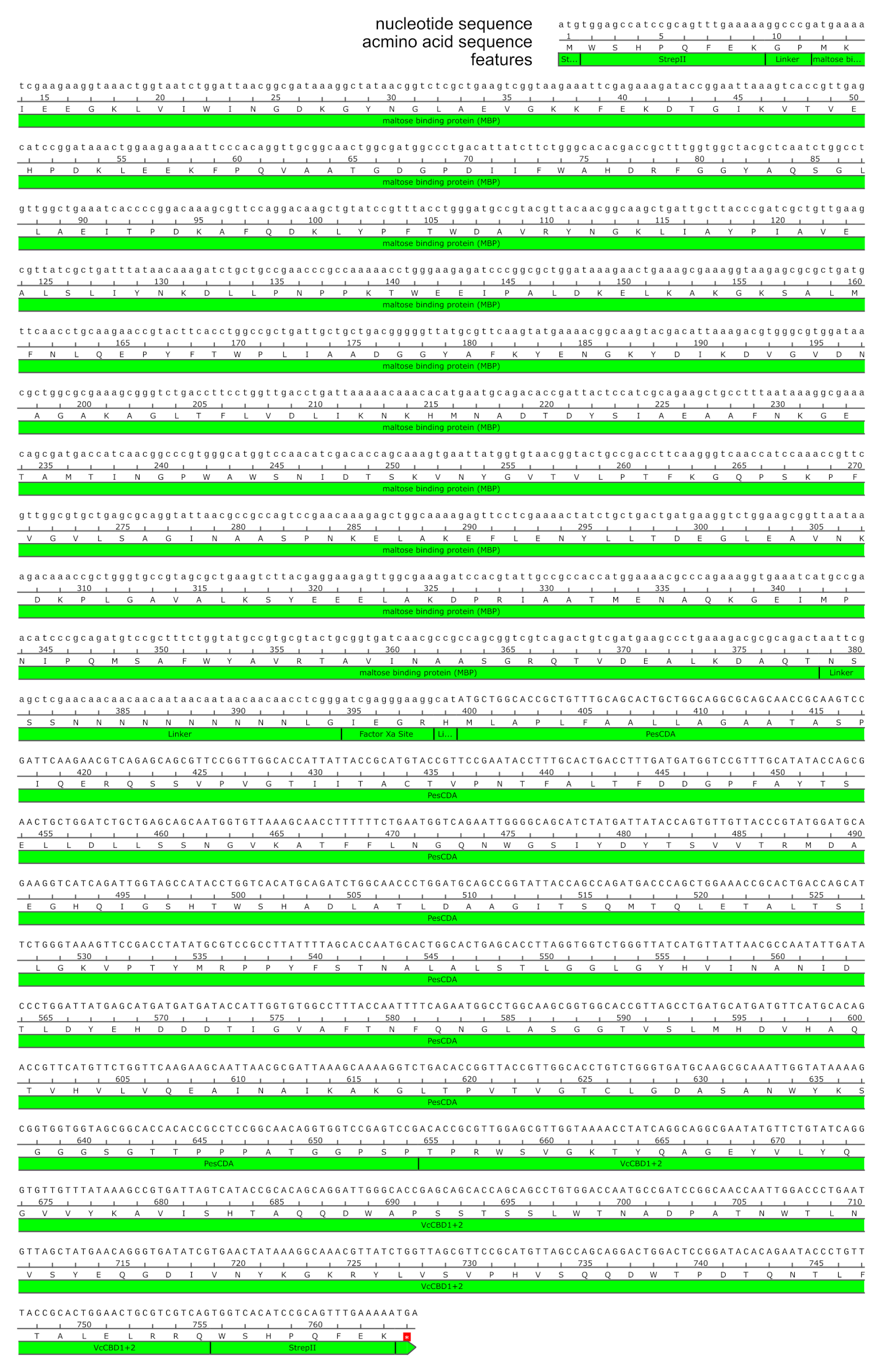

Supplement: S12 Fig — All features are annotated in the green underlying box. (TIFF) [file pbio.3002459.s012.tiff]
