# Supplementary material for: Engineering of a chitin deacetylase to generate tailor-made chitosan polymers
Source: PLoS Biol. 2024 Jan 18;22(1):e3002459. doi: 10.1371/journal.pbio.3002459 (PMC10796014; doi:10.1371/journal.pbio.3002459)
Supplement: Attachment — Submitted filename: ResponseToReviewers.pdf [file pbio.3002459.s018.pdf]

REVIEWS:

Reviewer #1: This manuscript by Bonin et al presents a study on the amino acid residues that comprise the substrate binding subsites of a chitin deacetylase to inform the generation of tailor made variants with altered properties. A combination of protein engineering and theoretical considerations involving extensive computer modelling was used to analyze the chitin deacetylase from Pestalotoipsis sp. The study identified several amino acids that would appear to confer substrate specificity and this was used to engineer enzyme variants with apparent enhanced activity toward specific substrates. The manuscript is organized logically and written clearly - it is concise while presenting all pertinent information. The interpretation of the data based on homology modeling, substrate docking and molecular dynamic simulations in combination with the generation and analysis of 513 muteins (470 verified by sequencing) is logical. That said, a major issue that the authors need to
address is their use of (more or less) qualitative data regarding the "activity" of the muteins produced. The activity levels observed and subsequently discussed do not appear to be specific activities, which would account for the concentration of enzyme present. The specific activity of a mutein would directly report on the effect of an amino acid replacement, and thus identify critical residues, in this case for substrate binding. However, the activities reported do not account for enzyme concentration and the assumption is that each mutein studied is produced at the same level. Of course, with 470 muteins produced, determination of specific activities would require significant effort, but some sense of enzyme production would be needed to support the interpretations made as currently stated. Without these analyses, the authors need to acknowledge this shortfall with their data, and discuss it accordingly.

Thank you for the good feedback. It is correct that we did not measure the specific activities of all muteins because this, as you said, requires significant effort and is probably error prone on that scale especially because of the magnetic beads which make any photometric enzyme detection difficult. However, we were acutely aware of this issue. So instead of quantifying the enzyme concentration, we addressed this important point in preliminary trials which we have not described in detail in the first version of our manuscript. In this revised version, we now added this information. We highlighted the fact that enzymes were purified using optimized small amounts of chitin-coated magnetic beads so as to saturate the beads and, thus, ensure similar enzyme concentrations in all wells. We tested different dilutions of the beads using the PesCDA wild type and settled on a 1:10 dilution. As an activity increase is still visible with the lower 1:5 dilution, we expect that the beads are saturated with our enzymes if we use a 1:10 dilution (see Fig. S1). Of course, we agree that this cannot completely ensure saturated beads for all muteins as some of them might be produced in strongly reduced amounts compared to the wild type, as also stated in the beginning of the results section.

However, we have further evidence to trust our results. When a student in our group screened the subset of muteins, she forgot to use the autoinduction components for the growth medium. Thus, in her screening, the *E. coli* strains expressing the different muteins showed strong differences in their cell density visible as the different sizes of the cell pellets (see figure below). We think we can safely assume that as a consequence, these cultures largely differed in the amounts of enzymes produced. Still, when we repeated the screening using the proper autoinduction medium, the resulting activities showed the same pattern relative to each other as seen before in the experiment without induction. We should add that we never expected such a screening to reliably detect small differences in the activity of muteins, such as 60 % versus 80 % of the wild type's activity. This is why we stated that the results were evaluated carefully, making sure to avoid relying on the activity levels of a single mutein without further rationalization. We emphasized that we focused on the overall activity pattern for each position, which remains the same even with strong differences in cell density as mentioned above. For the sake of brevity, this may have become too cryptic to understand for the reader and we tried to elaborate more on this aspect in this revised version.

When presenting and discussing the results for the HPLC-MS based screening of seven selected positions, we indeed do discuss single muteins or activity values for a group of muteins. However, even here, we tried to never base our conclusions solely on a single mutein's activity but on the activities exhibited by several muteins. In addition, our conclusions are also based on the MD simulations.

Finally, even if enzyme concentrations would be determined, one could not distinguish between correctly and incorrectly folded proteins. Thus, a mutein might show very similar enzyme concentration compared to the wild type, while its activity is strongly reduced. Even when using known enzyme concentrations, we could not distinguish whether the reduced activity results from a missing interaction with the substrate or whether the protein is simply folded incorrectly.

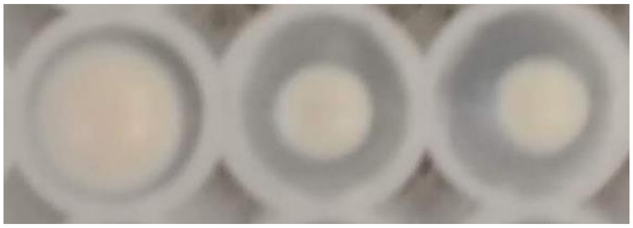

Different sized *E. coli* cell pellets after centrifugation of cultures grown in medium excluding the autoinduction components.

Reviewer #2: This manuscript by Bonin et al. presents an extensive site-directed mutagenesis campaign to alter the specificity of a chitin deacetylase toward individual N-acetyl groups on chitin oligosaccharides. The results represent a large amount of work, including the production and assay of site-saturation mutants of over two-dozen amino acid positions, followed by further refined study. The manuscript is very well produced regarding writing and data representation and the conclusions are well-supported.

However, I do not recommend this manuscript for publication in PLoS Biology. Fundamentally, this is a classical protein engineering study, which would be more appropriate for a field-specific biochemical or biotechnology journal. From the perspective of PLoS Biology, this study really provides no fundamental insight into biological processes. Conceptually and methodologically, the study generally uses well-worn protein engineering and analysis approaches. Likewise, the selection, engineering and application of chitin deacetylases to produce specific oligosaccharide acetylation variants has also been explored, notably by this group (e.g. refs. 20, 27, 30) and others. Notwithstanding the comments in the first paragraph above, it does not seem that this study meets the bar of "works of exceptional significance, originality, and relevance in all areas of biological science".

Thank you for the positive feedback regarding the amount and quality of our work as well as our presentation of our data and conclusions. Regarding the missing insight into biological processes, we would like to disagree. This study gives detailed insights into the substrate recognition of a carbohydrate active enzyme which is relevant in many areas of biology. To our knowledge, a screening of a defined mutant library on this scale with different substrates, time points and detection methods (photometric, MS1, MS2) is not commonly done. Especially not when combining all these data with detailed *in silico* studies. Of course, the selection and application of chitin deacetylases to produce specific chito-oligosaccharides was done before and is an ongoing process to enlarge the toolbox of known CDAs to produce more and more specific chito-oligosaccharides. However, targeted CDA engineering is still at an early stage, arguably because of the so far limited understanding of the determining factors that facilitate different binding modes. We would argue that this paper is a big step forward in understanding these determining factors and that these insights can also be applied to other closely related, but probably also some less closely related carbohydrate active enzymes. To strengthen this applicability to other enzymes, we included several spreadsheets in our supporting material containing screening activity, MS1 and MS2 values as well as all values calculated from our MD simulations so that others can have a detailed look at the results and e.g. find all values for homologous positions in their enzymes.
In addition to these considerations, we would like to highlight that a plethora of research articles highlight the potential of chitosan for a lot of applications. However, it was shown that the reproducibility of these results is rather low if different chitosans are used. Therefore, the use of specific chitosans with reproducible properties is very important. These properties include the pattern of acetylation as shown in a recent publication by Sreekumar et al. (DOI: 10.1038/s41467-022-34483-3). As shown in their paper, CDAs can be used as a tool to create these tailor-made chitosans. Unfortunately, we are not yet able to produce all desired patterns of acetylation. With the results presented here, we showed for the first time that we can actually mutate a CDA to

alter the final pattern of acetylation.

Reviewer #3: In their article Bonin et al. use site saturation mutagenesis to investigate the role of active site amino acid residues play in catalysis for the Pestalotiopsis sp. chitin deacetylase. These muteins were investigated through screening with a fluorogenic detection reagent, and through mass spectroscopy analysis of released products. The authors give some interesting insight into the role of H199 muteins in catalysis and characterize these mutants in both N-deacetylation and N-acetylation assays.

Although the authors do show differences in activity for various mutants, the conclusions that they draw from these experiments are confounded by the lack of enzyme quantification performed. The decrease (or increase) in any activity observed in the many assays performed in this work may be either a result of genuine changes in the catalytic rate or a change in purification yield – that may be due to stability changes, codon usage or protein folding rates. The conclusions drawn from the screening experiments are therefore difficult to support, and their discussion should be curtailed.

First, we would like to thank reviewer #3 for the thorough review of our paper. We tried to address all points where possible or otherwise tried to rationalized our decision why we opted to keep the original version. We have addressed the main concern about the missing enzyme quantification in our answer to the question for "p. 13, L. 295" below, but see also our answer to reviewer #1 above who had expressed the same concern.

Major issues:

p. 7, L. 140 The arbitrary choice of outliers makes the quality of the data dubious. There are statistical tests that can be performed to identify outliers, and these should be used in place of the automated 'outlier' removal criteria.

We understand the concern which is raised here. The screening was done in part using a pipetting robot, which could introduce errors (e.g. a fresh pipetting tip not being taken up properly). These errors can easily be corrected if done by hand, or corresponding wells can be marked and repeated. This was not possible for the parts taken over by the pipetting robot. Because of these observations, we wanted to include an outlier detecting mechanism. This mechanism was supposed to filter out the values which clearly deviate from the other replicates, however, this should not be done manually but using a simple algorithm. We included a spreadsheet with all values with and without the outlier elimination so the reader can compare the two versions. Of course, there are statistical tests for outlier detection, but these are not intended to be used on only four replicates. We based our decision on a detailed overview about outliers and their detection by Jim Frost (https://statisticsbyjim.com/basics/outliers/, see "My Philosophy about Finding Outliers")

P5, L80 The authors should justify why swiss-model was used instead of Alphafold2. They should also state whether any of the amino acids investigated here have large changes in positioning in the alfafold2 model.

When we started working on PesCDA, Alphafold2 was not published yet. Because CDAs are structurally very conserved in their core, swiss-model usually results in very good predictions as well. Still, we compared the two models as suggested. The RMSD between the 27 amino acids included in the library for the two models is 0.78 Å. A corresponding figure was added to the supporting information.

p. 12, L. 286 Introducing the CBDs from Vibrio cholerae CDA results in a chimera protein. Have the authors investigated the effect of these CBDs on enzyme activity? The CBD and the catalytic domains may interact, therefore the effects of introducing these domains from one homolog into another should be quantified and reported. Furthermore, as this is a chimeric protein it is inappropriate to refer to this protein as the 'Wild-type'.

We did not specifically test if the two domains interact with each other. There are several reasons why we did not test this. According to the structure predictions (swiss-model but also Alphafold2), the CBD from PesCDA, which was exchanged for the VcCDA CBD, is attached at the backside of the enzyme, exactly opposite to the substrate binding site. With the rather short linker between the two domains, it is highly unlikely that the

domains interact in a way that would affect the PesCDA substrate binding site.
PesCDA was studied before with its own CBD and our results (e.g. concerning the generated pattern of acetylation during oligomer deacetylation or polymer *N*-acetylation), i.e. when using the enzyme with the Vibrio CBD, match the ones previously published, i.e. with the enzyme containing its own CBD (see Cord-Landwehr et al. (DOI: 10.1038/srep38018) and Sreekumar et al. (DOI: 10.1038/s41467-022-34483-3).
During our screening, the enzymes were still attached to the beads via the CBD. Thus, it is even less likely that the CBD could interact with the PesCDA, influencing its catalytic activity.
In our initial submission, we stated in the corresponding material and methods section (p. 6, L 109) that we refer to the fusion construct as PesCDA WT to avoid any confusion. We tried to rename PesCDA WT to PesCDA control, however, this seemed to be very confusing in the context of the paper. Because we only discuss the CDA domain which is indeed the wild type domain, we think keeping the WT nomenclature during the paper is less confusing for the reader.

p. 13, L. 295 Using chitin affinity to ensure similar enzyme concentrations while using a CBD would be fine if you weren't also modulating the enzyme's affinity for chitin by mutating the residues that can modify the chitin on purification beads. Therefore, it appears improbable this purification method results in a similar interaction pattern with the beads. To quantify differences in enzymatic activity for the muteins you should quantify the purified enzyme present in you experiments. Especially since you mentioned the possibility of low expression yields being a concern (p. 14, L. 304). This might occur due to folding problems being introduced from mutating the primary sequence of the protein, as you also note at p. 16, L. 349, or it may simply be due to one protein expressing better than another.

During the step in which the enzymes are bound to the chitin magnetic beads (CMB), the samples are cooled to 4 °C, strongly reducing the enzyme activity (temperature optimum at ~60 °C, see Cord-Landwehr et al. (DOI: 10.1038/srep38018)). Furthermore, as also shown by Cord-Landwehr et al., the PesCDA activity is very low on α- and β-chitin which, given that this has been observed with all CDAs investigated so far, is not expected to change dramatically with a single amino acid mutation. Hence, we think it is highly unlikely that the enzymes will modify the CMB under these conditions. To avoid any of these concerns, we tested other beads to which the enzymes bind via Strep-tagII; however, these tests resulted either in very low yield and therefore activities, or in high standard deviations resulting from difficulties of pipetting these beads. This is why we opted for the CMBs because we concluded that any potential draw backs from purifications with CMBs will not strongly influence our results, and these were the beads we could pipette most reproducibly.
CMBs which are present in the sample make a reliable photometric detection of enzyme concentration impossible. Here, an elution step would be required which would increase the complexity of the screening method. Because of the reasons we outlined above in our reply to reviewer #1, we think that our method of adjusting the enzyme concentration by saturating the CMBs is sufficient. In this way, we have an upper boundary. You are right that we cannot distinguish between a low enzyme yield, incorrectly folded or poorly active enzymes, which we now highlighted further in the beginning of the results section. If we were to measure enzyme concentration, we could still only detect a low enzyme yield whereas we still could not differentiate between incorrectly folded or poorly active enzymes. In the end, this is also not the aim of our study. If a specific single point mutation repeatedly leads to a reduced enzyme activity (independent of the underlying cause), we assume that a different amino acid is required at this position for a correctly working CDA.

p. 14, Fig 2. These images are not easy to interpret due to the font being very small. Consider moving the numbers to the SI, combining with the SD and p-value for a clearer overview. It is also not clear which results (if any) are significantly different from the unmutated protein. Without the significance of the results it is difficult to make any meaningful conclusions from this data.

We agree that the numbers are small. But we think the numbers are important as the help to compare non-neighboring boxes with seemingly similar colors, because our eyes detect colors differently depending on surrounding colors. All figures (except fig. 1) are vector graphics so they can be zoomed in without losing quality to take a closer look at the numbers if needed.
We have now included p-values calculated from a two-sided t-test assuming unequal variance in the "FullA4screening_with(out)_outliers" spreadsheet. However, we would like to caution that with only four

repetitions, giving p-values can give a wrong level of confidence. Just by chance, some positions may have very similar values in all replicates, resulting in a low p-value, whereas the values obtained for other positions may differ more strongly, resulting in a higher p-value. Still, if both cases would e.g. have a mean of 0.8 we would not trust one value more than the other, because we assume that much of the variation may come from the screening itself and not from the muteins.
As highlighted in the beginning of the results (p. 13, L. 301), we do not want to rely on activity levels of a single mutein without further rationalization. As an example, we often compared e.g. aromatic vs. non-aromatic amino acid exchanges. Here, p-values for a comparison with the unmutated protein are not helpful.

p. 15, L. 323 This explanation does not account for the possibility that this mutant facilitates a different binding mode. To claim it is a false positive you should do more experiments to test this assumption. For example, re streak the clone, verify the plasmid sequence, quantify the protein being purified and test the activity again.

You are completely right with this concern. The first screening was done in the lab of Prof. Planas with a copy of the SSM library. We assume that a wrong colony (possibly P138G which was next to the R137V well) ended up in the well where R137V should have been. Of course, we tried to sequence the plasmid but despite many tries, this was not successful. So, without any confirmation that the plasmid is wrong, we didn't want to remove this mutein. Instead, we left it in to use it as an example to show that this can happen with a screening on this scale. The avoid any claim, we changed to wording from "[…] can be assumed to be a false positive result […]" to "[…] is possibly a false positive result […]".

p. 24, L. 536 This sentence refers to an image that indeed shows more products being formed after 48h. There is a lack of explanation to later refer to this as a discussion on it (p. 26, L. 625). Why does this happen? Could it be explained by WT product inhibition or re-acetylation of GlcN being more pronounced than it is for these muteins?

You are right that this cannot be referred to as "as discussed before". We changed the wording at this place. CDAs are able to *N*-acetylate GlcN residues, however, only if very large amounts of acetate are present (we used 1.5 M sodium acetate buffer for our polymer *N*-acetylation). In the oligomer screening, we used 0.5 mM A4 which results in a 0.5 mM acetate release for the WT, which should not result in a significant *N*-acetylation. It is also not expected that these amounts of acetate inhibit the enzyme. The PesCDA inhibition by acetate was tested in Cord-Landwehr et al. (DOI: 10.1038/srep38018), where they showed that with 5 mM acetate present, the PesCDA still retains 98.7 % of its activity (with roughly double the amount of A4 substrate). Consequently, we assume that the WT can be inhibited by its own AADA product or that other binding modes are highly unlikely due to its strong preference for binding mode [-2, +1]. We discussed the strong preference for this binding mode in the discussion starting at p. 32, L. 741. The possibility of product inhibition is further discussed in the following starting at p. 34, L. 776.

P. 18 Fig.3 It is inappropriate to report SD values with so many significant digits as it gives a false impression that you know how much SD there is. Values should be only be reported with significance limited to the SD. For example. Your value of -27.01 +/- 11.70 should be reported as '-30 +/- 10' . Also, your value of 0.05 +/- 0.00 gives the false sense that there is no SD, you should report this as 0.050 +/- 0.002 or whatever values you have calculated for this.

We changed the small values to include three decimal places. For the values with the larger SDs, however, we want to keep the values the way they are to avoid any confusion for the reader. We understand your point, as reporting a value to a certain precision with a correspondingly high standard deviation usually seems wrong because the used method seems to be inappropriate to give a value with such a high precision. However, the data shown in fig. 3 is calculated from MD simulation which typically calculate everything with a higher precision then those given by our results. So, the high standard deviation in this case actually comes from the simulation itself in which the substrate fluctuates more strongly in this binding mode at this subsite. Therefore, the BFE value which is here calculated for every 100th frame in the simulation differs more strongly because the GlcN positions relative to the surrounding amino acids differ more strongly between the different frames. In the end, the large standard deviations for the BFE of a GlcN unit being placed at subsite 0 for

binding modes [-3, 0] and [-1, +2] highlight this stronger fluctuation. This can be linked to what you mentioned earlier about product inhibition. If we compare these results to the ones obtained for AngCDA in our previous paper, it seems that PesCDA could indeed be capable of binding AADA in binding mode [-2, +1], leading to product inhibition.

P. 17/18. Please include a note on the correspondence between the docking calculation methods that you have used and their experimental calculations of binding energy. This will help the reader evaluate the significance of the values determined here.

In fact, we did not determine the binding energies experimentally. Even if experimentally determined binding energies were available, these could only be compared to a limited extent to the calculations based on the MMGBSA approach. This method is not intended to yield realistic kcal/mol numbers but instead yields BFE values that can be compared between different simulations using a similar setup. There are methods to calculate more realistic kcal/mol values, which are however computationally much more demanding. The MD simulations were also not intended to yield realistic BFE values, but should give an insight into the underlaying molecular interactions to narrow down the most interesting positions for further screening and to make sense of our in vitro results. We further highlighted this consideration about the MMGBSA approach in the beginning of the chapter. For those readers interesting, we referred to our recent paper about another CDA (see Bonin et al., DOI: 10.1016/j.jbc.2021.101129), where we used the same MD simulation approach and discuss the comparison of in silico and in vitro results in detail.

P.20 The results of the docking experiments are discussed as though they are experimentaly determined interactions and energies. For example, on line 460 : ‘ the side chain nitrogen and oxygen form hydrogen bonds with the -1 sugar in binding mode …’. This statement has however not been experimentally validated; this is a hypothesis.

To avoid any confusion here, we included a note in the beginning of this section stating “In the following we will highlight some of the observations from our MD simulations and relate them to the calculated RMSF and BFE values. “

P. 20 The term ‘stack’ is repeatedly used to refer to interactions between aromatic acid amino acids and sugar residues. This term is ambiguous, and it makes the reader think of pi-pi stacking interactions, which clearly cannot be present with the sugar residue. If the authours intend to state that there are supposed CH-pi interactions, they should calculate whether these are within the proper distances and at the proper angles to be true interactions.

We referred to this interaction as “stacking” based on a paper by Asensio et al. from 2013 called “Carbohydrate-Aromatic Interactions” (DOI: 10.1021/ar300024d). We agree, however, that this can lead to confusion, so we now refer to this interaction as carbohydrate/aromatic interactions or CH/π bonds. In some cases, we still describe the 3D orientation of the sugar and aromatic ring as “one stacks against the other”, but we avoided this where possible. Other than hydrogen bonds which are typically analyzed in MD simulation by a simple distance and angle cut-off, there is, to our knowledge, no widely used way to determine if CH/π bonds are present. As stated in the aforementioned paper: “Indeed, a geometry analysis performed over an extended set of experimental sugar protein complexes showed that the position of the center of the pyranose ring can take a large set of spatial orientations relative to the aromatic residue.[25] As it will be mentioned below, in the Theoretical Evidence section, there is a weak orientation dependence of the sugar aromatic interactions. Therefore, these complexes display a high dynamic character.”
In fact, we found this paper while searching for a method to quantify carbohydrate/aromatic interactions in our MD simulations. Because we could not find a suitable method, we had to visually evaluate if the aromatic residue and the corresponding GlcNAc or GlcN unit stay in a rather fixed orientation towards each other. This was the case for those interactions which were here referred to as ‘stacking interactions’.

Minor Issues:

p. 6 , L. 105 Why did you choose to test the activity on an A5 substrate when the rest of the research appears to have been focused on the A4 substrate?

This section only refers to an initial activity test looking for a yes/no answer for which the pentamer was well suited (admittedly, the tetramer would have worked almost as well). We later switched to A4 because we can produce the different corresponding mono-deacetylated substrates (which were not available for the pentamers) which were used later in the detailed screening.

p. 10, L. 204 Discuss here why are only these 2 mutants were used in the experiment, based on what metric were these selected? Mentioning this in the last part of your results and discussion is to late.

We have added the explanation that the muteins tested here showed an increased and decreased GlcNAc preference at subsite -1 during our SSM library screening.

p. 12, L. 272 Following the A = GlcNAc and D = GlcN labeling and your explanation that A4 is a GlcNAc tetramer, the D46 could refer to a 46-mer of GlcN instead of aspartic acid 46. Updating the carbohydrate abbreviations is advised to avoid confusing amino acids and sugars.

As we only used small oligomeric substrates or large polymeric substrates, we assumed that it is unlikely that someone would confuse D46 for a 46-mer of GlcN. In fact, the abbreviations GlcNAc = A and GlcN = D are commonly used in literature describing chitin and chitosan modifying or hydrolyzing enzymes such as CDA. Thus, we prefer to keep this labeling. Alternatively, we could label D46 as Asp46 but then, we would need to change all amino acid names. This was actually discussed internally before, but we decided to keep the one letter code because these amino acids are so commonly referred to and discussed in our paper.

p. 13, Fig 1. Colour-coding the signal peptide and substrate both green is inconsistent and confusing. Could be improved by adding another colour for the signaling peptide.

Thank you for the suggestion. We changed the color of the signal peptide.

p. 22, L. 498 You should specify the 'type of sugar' to the acetylation state of the glucosamine. Written like this you suggest that any sugar goes.

As stated in the referenced sentence, the hydrogen bond, which is mentioned here, is not formed with the acetyl group. Thus, it does not facilitate a preference for a GlcNAc unit, but instead it can be formed with both a GlcNAc and GlcN unit. We replaced the ‘independent from the type of sugar present’ with ‘independent from whether a GlcNAc or GlcN unit is present’.

p. 27, L. 631 Could this be accounted for by protein folding deviations when the hydrophobic pocked isn't formed?

Yes, you are right, the protein could fold differently if the hydrophobic pocket isn’t formed. One could imagine that this leads to a more restricted +1 subsite and thus, AADA is preferentially further deacetylated to AADD instead of ADDA. We included your suggestion and mentioned that folding deviations could result in a similar effect. However, we assume that this shift is rather caused by the missing solvent exposure of F141 because we also saw this effect in our MD simulations (fig. 4). Here, the BFE of F141 is reduced in binding mode [-3, 0] compared to the other binding modes.

p. 41, figure Needs replicates.

This experiment only shows the *N*-acetylation of polyglucosamine. The produced polymers of comparable F_A’s were then used to test their average A- and D-block sizes. The *N*-acetylation was only done once because a repetition would never result in the same F_A polymers. Thus, results from different *N*-acetylations could not be directly compared.

p12, L. 268 consider the term docked instead of ‘placed.’

Well spotted. We changed ‘placed’ to ‘docked’.

p24. L 543 the term ‘catchup’ is ambiguous, consider using better terminology.

You are right. We were also not 100% happy with the term ‘catch up’. We discussed this again an changed it to ‘converge towards’.

P23. Figure 6. These charts would be more interpretable if expressed as rates, either as rates of substrate decay or as initial rates where appropriate.

We considered changing the scale of this heatmap, e.g. giving the relative acetate release as done in fig. 7. We assumed that this would be misleading because the data from the first screening (fig. 2) is given as values relative to the WT (which cannot be changed due to the way these values are calculated). To make the values of fig. 6 more interpretable, we added fig. 7 in which we show the relative acetate release for selected muteins.

P 29 L 683. Presence of a positively charged amino acid H199 lead to charge-charge repulsion with a deacetylated glucosamine residue? Is this why you the specificity pattern

For some reason, it seems that a part of the second sentence was lost during the review process. The possibility of charge-charge repulsion with H199 is further discussed in the discussion starting on p. 34, L. 791. We cannot say at which rate H199 or glucosamines in our substrates are positively charged at pH 8.5, but we can assume that this rate increases towards a lower pH. And indeed, when Hembach et al. tested PesCDA deacetylation on A4 at pH 7, they showed that even higher amounts of ADDA instead of AADD are produced. So, we can conclude that charge-charge repulsion probably plays a role here, however, because of the unknown protonation rates at pH 8.5, we cannot estimate the importance of this repulsion at our screening conditions.
