# Supplementary material for: Engineering of a chitin deacetylase to generate tailor-made chitosan polymers
Source: PLoS Biol. 2024 Jan 18;22(1):e3002459. doi: 10.1371/journal.pbio.3002459 (PMC10796014; doi:10.1371/journal.pbio.3002459)
Supplement: Attachment — Submitted filename: ResponseToReviewers2.pdf [file pbio.3002459.s019.pdf]

Reviewer remarks:

Reviewer #1: My issue has been adequately addressed.

Reviewer #3: Regarding the WT nomenclature of PesCDA. The construct described in the paper is not a wild-type protein, it is a fusion construct. Naming it as a wild-type protein, no matter what the reasoning behind it is factually incorrect. It is not a wild-type protein.

We addressed the problem with the WT nomenclature by changing it to $PesCDA^{nm}$ (non-mutated PesCDA) throughout the text, in the corresponding figures and supporting materials. We only kept the WT nomenclature if we did not refer to the whole enzyme but to individual amino acids.

A method to determine whether CH/pi interactions are within reasonable distances and angles can be found in https://doi.org/10.1021/jacs.5b08424 by Hudson et. al. See Figure 3. The author should apply these calculations to CH-pi interactions that they 'visually evaluate'.

We had a look at the provided article, which we had not found before. Thank you for providing this information. Still, we did not calculate whether these interactions are indeed CH/pi interactions according to these cutoff values for all simulations, because this would be out of the scope of this article. One aspect which we think makes this a strong article, is the combination of our *in vitro* and *in silico* data. Whereas the *in silico* approach might not perfectly reproduce if these are in fact CH/pi interactions, our *in vitro* data show that the aromatic nature of the three relevant residues is indeed crucial for enzyme activity. Therefore, to avoid any further confusion, we removed the term CH/pi interactions completely and only referred to these as carbohydrate/aromatic interactions.
